# Thorax morphology of selected species of the genus *Cacopsylla* (Hemiptera, Psylloidea)

**DOI:** 10.3897/zookeys.319.4218

**Published:** 2013-07-30

**Authors:** Jowita Drohojowska, Małgorzata Kalandyk-Kołodziejczyk, Ewa Simon

**Affiliations:** 1 Department of Zoology, University of Silesia, Bankowa 9, 40-036 Katowice, Poland

**Keywords:** Psylloidea, *Cacopsylla*, morphology, thorax

## Abstract

The paper concerns with characteristics of a thorax morphological structure *Cacopsylla* Ossiannilsson, 1970 species, referring to an analysis of five species classified in the past in three subgenera. The structure of the sternites, tergites and pleurites of all the parts of the thorax was studied by a scanning microscope. Descriptions of particular elements building up thorax plates, their shape, size and links as well as a course of all the clefts and sulcus are provided. The study of thorax morphology of *Cacopsylla* species suggests that the thorax morphology is relatively homogenous within a genus.

## Introduction

Jumping plant-lice or psyllids (Psylloidea) constitute a superfamily of Sternorrhyncha comprising over 3800 described species of small plant-sap feeding insects (Li 2011).

Studies of the adult morphology have concentrated mainly on the head, fore and hindwings as well as the terminalia (Klimaszewki 1975, Hodkinson and White 1979, Ossiannilsson 1992). Despite the fact that the thorax of adult psyllids is relatively large compared to the total body length, there is surprisingly little information available on its detailed morphology (Stough 1910, Crawford 1914, Taylor 1918, Weber 1929, Tremblay 1965, Ouvrard et al. 2002, 2008). This is mainly because most morphological data relate to diagnostic features of which few have been found on the thorax. Heslop-Harrison (1951) sought taxonomically useful characters in the prothorax (shape of propleurites and propleural sulcus) to separate his subfamilies Liviinae and Aphalarinae. Loginova (1978) used the relative lengths of the pronotum, mesopraescutum and mesoscutum to separate *Thamnopsylla* from the other subgenera of *Cacopsylla*. This character was also used by Burckhardt (2010). Klimaszewski (1975) used the shape of propleural sulcus to separate Arytaininae from Psyllinae.

The genus *Cacopsylla* Ossiannilsson, 1970 belongs to the subfamily Psyllinae (Psyllidae) and consists of approximately 170 described species associated with woody dicotyledonous plants as does the majority of species of this family and psyllids in general. The genus has a predominantly Holarctic distribution with a few species occurring in the Afrotropical, Oriental and Australian biogeographical regions (Hollis 2004, Burckhardt pers. comm.). This large genus has been divided into the taxa *Cacopsylla* s. str. Ossiannilsson, 1970, *Hepatopsylla* Ossiannilsson, 1970, *Osmopsylla* Loginova, 1978 and *Thamnopsylla* Loginova, 1978. Recently Burckhardt and Ouvrard (2012) synonymised all subgenera with *Cacopsylla*.

## Material and methods

Adult psyllids were collected with an entomological sweep-net and killed in vapours of potassium cyanide. After air drying and removing the wings and legs, the specimens were cleaned with 10% alcohol and then mounted on a stub for the analysis in a low vacuum electron scanning microscope S-3400N. The specimens were not gold coated. The following species were analysed: *Cacopsylla peregrina* (Foerster, 1848), syn. *Cacopsylla (Cacopsylla) peregrina* (Foerster, 1848); *Cacopsylla sorbi* (Linnaeus, 1767), syn. *Cacopsylla (Cacopsylla) sorbi* (Linnaeus, 1767); *Cacopsylla ambigua* (Foerster, 1848), syn. *Cacopsylla (Hepatopsylla) ambigua* (Foerster, 1848); *Cacopsylla crataegi* (Schrank, 1801), syn. *Cacopsylla (Thamnopsylla) crataegi* (Schrank, 1801); *Cacopsylla melanoneura* (Foerster, 1848), syn. *Cacopsylla (Thamnopsylla) melanoneura* (Foerster, 1848). Additional 24 *Cacopsylla* species deposited in the Department of Zoology, University of Silesia (Poland) were analysed using Nikon MZ1500 stereoscopic microscope.

Morphological terminology and the list of abbreviations used to describe the photographs is after Ouvrard et al. (2002, 2008): aas- anterior accessory sclerite, acl2- anapleural cleft, apwp- anterior pleural wing process, axc2- axillary cord, cx1- procoxa, cx2- mesocoxa, cx3- metacoxa, epm1- proepimeron, epm2- mesepimeron, epm3- metepimeron, eps1- proepisternum, eps2- mesepisternum, eps3- metepisternum, fpa2- fossa of the pleural apophysis, ftna2- fossa of the trochantinal apodeme on mesothorax, ftna3- fossa of the trochantinal apodeme on metathorax, kes2- katepisternum, mcs- meracanthus, nt1- pronotum, pas- posterior accessory sclerite, pls1- propleural sulcus, pls2- mesopleural sulcus, pls3- metapleural sulcus, pnt2- mesopostnotum, pnt3- metapostnotum, ppt- parapterum, psc2- mesopraescutum, ptm2- mesothorax peritreme, ptm3- metathorax peritreme, s2- mesosternum, sc2- mesoscutum, sc3- metascutum, scl2- mesoscutellum, scl3- metascutellum, tg- tegula, trn2- mesothorax trochantin, trn3- metathorax trochantin.

## Results

In all *Cacopsylla* species the head is strongly inclined from longitudinal body axis resulting in an arched dorsal outline of the thorax. The prothorax is the smallest thorax segment and undergoes strong modifications because the mouth parts are displaced posteriad. Mesothorax is the biggest part of the thorax related to a functional dominaton of forewings, which is further connected with the development of muscles that move them.

**Figure 1.**
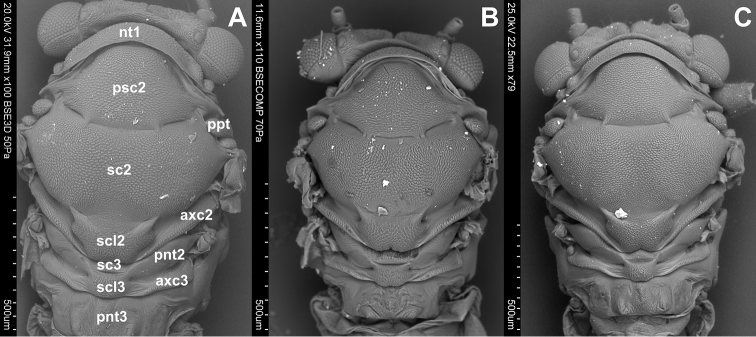
Dorsal side. **A** *Cacopsylla peregrina* **B** *Cacopsylla crataegi* **C** *Cacopsylla ambigua*

Dorsum (Figs 1A, B, C). The dorsum of the prothorax consists of one sclerite, the pronotum (nt 1), which is narrow and arcuate medially. Anterior and posterior margins of pronotum are distinct. The pronotum is narrower than the head including eyes but wider than vertex. Laterally the pronotum is as long as the pronotum along midline. The dorsum of the mesothorax is divided into the large mesonotum and the small, transverse, weakly raised mesopostnotum. The mesonotum is divided into three sclerites: mesopraescutum (psc2), mesoscutum (sc2) and mesoscutellum (scl2), which are separated from each other by a distinct sulcus. The mesopraescutum (psc2) is a medium sized sclerite, it’s fore margin is slightly arched and covered by the hind margin of the pronotum. The hind margin of the mesopraescutum is vaguely semicircular. The mesopraescutum and mesoscutum are linked together because the sulcus which separates them is not complete. In the middle there are two symmetrical joints except for *Cacopsylla ambiqua*. The mesoscuctum (sc2), the biggest sclerite of the mesonotum, is U-shaped with elongated processes reaching the parapteron. The mesoscutellum (scl2) is small and trapezoidal. The hind part of the mesoscutellum is strongly incised in the middle in *Hepatopsylla* and some *Thamnopsylla* species. The mesopostnotum, situated behind mesonotum, is not visible dorsally as it is covered by the mesoscutellum (scl2), laterally it forms a narrow ridge running diagonally towards the mesoepimeral sclerite. The metathorax consists dorsally of the metanotum and metapostnotum. The metanotum consists of two plates, the metascutum (sc3) and the metascutellum (scl3). The metascutum (sc3) is a small sclerite, while the metascutellum (scl3) is bigger and clearly raised in relation to the metascutum. The metapostnotum (pnt3) is relatively large and trapezoidal. The metapostnotum is completely fused with the first abdominal tergite.

**Figure 2.**
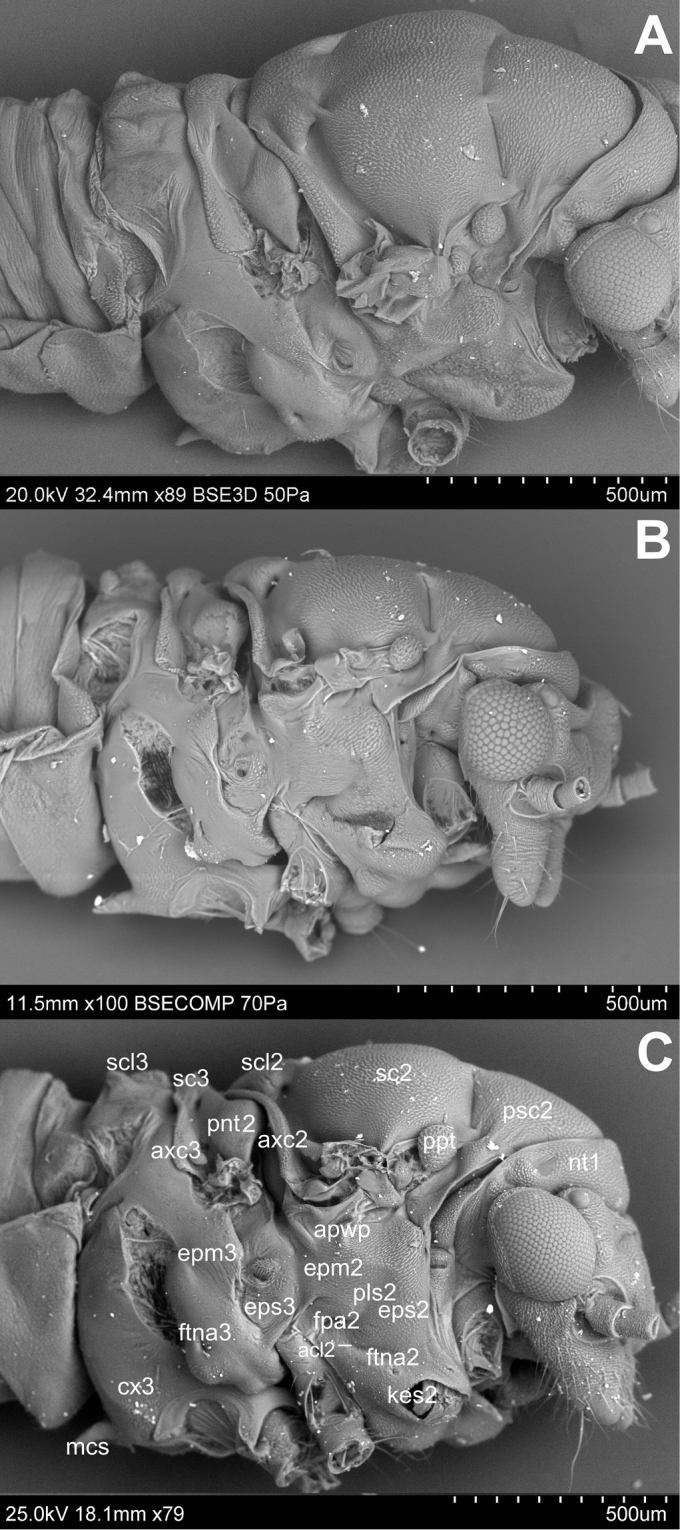
Lateral side **A** *Cacopsylla peregrina* **B** *Cacopsylla crataegi* **C** *Cacopsylla ambigua*

Pleura (Figs 2A, B, C). Laterally the pronotum is constituted by two easily visible pleurites (Fig. 4), the proepisternum (eps1) in front and the proepimeron (epm1) at the rear. Proepisternum and proepimeron are of the same size. They are always clearly separated from each other by the propleural sulcus, while their border with prothorax is not always visible. The propleural sulcus (pls1) runs from the basal coxal appendix up to the hind corner of the pronotum. The sulcus is straight without any curves. Between the pleurites of pro- and mesothorax, there are three tiny, well visible sclerites of arguable origin, which are called frontal and hind additional sclerites (aas and pas), and peritrema (ptm2) which surrounds the mesothoracic spiracle (Fig. 4). The mesothoracic pleurae consist of two large sclerites (Fig. 2), the anterior mesepisternum (eps2) and posterior mesepimeron (epm2). The shape of the mesepisternum (eps2) depends on the position of the pleural sulcus (pls2) and anapleural cleft (acl2). The mesothoracic pleural sulcus (pls2) is well visible and runs from the coxal condyle and approaches the middle of pleuron but does not reach the wing base. Shape and position of the mesopleural sulcus are variable within *Cacopsylla*. It is relatively straight in *Cacopsylla peregrina*, slighty curved in the distal part in *Cacopsylla melanoneura* or S-shaped and strongly curved in *Cacopsylla crataegi* and *Cacopsylla ambigua*. It forms a shallow furrow pleural with small, shallow fossa (ftna2) in *Cacopsylla melanoneura* or a distinct furrow with deep fossa in *Cacopsylla ambigua*. It is very long, reaching the end of the mesepisternum in *Cacopsylla crataegi* but considerably shorter in the other species. The anapleural cleft (acl2) is straight and almost horizontal, the transepimeral sulcus is very short and distinct, it is never straight. The anterior pleural wing process (apwp) is thin and elastic. Parapteron (ppt) and tegula (tg) have the same oval shape and the katepisternal complex (kes2) is rounded and subtle striped. The metapleurites are well developed. The metapleural sulcus (pls3) is visible and reduced at a short distance ventrad. This sulcus does not separate metaepimeron and metaepisternum entirely. The metapleuron is strongly modified by a vertical prolongation of the coxal meron. The latter enforces on metaepimeron and metaepisternum a development into a long, thickened arch stretching above the metacoxa. The metathoracic stigma (stg3), trochantin (trn3) and external fossa of the trochantinal apodeme (ftna3) are well visible.

**Figure 3.**
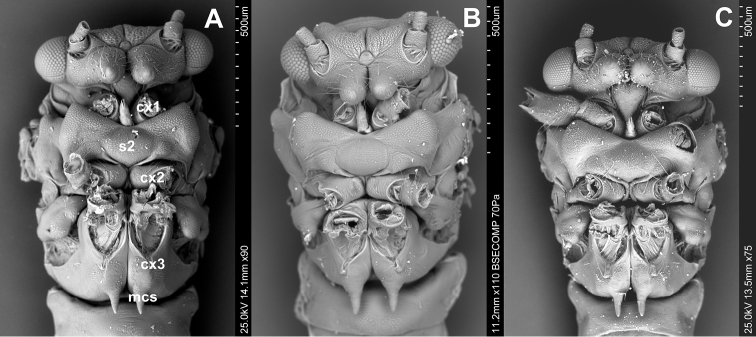
Ventral side **A** *Cacopsylla peregrina* **B** *Cacopsylla crataegi* **C** *Cacopsylla ambigua*.

Venter (Figs 3A, B, C). The prothoracic sternum of psyllids is significantly reduced and covered by the rostrum. The mesothoracic venter is constituted by a well-developed sclerite, the mesosternum (s2), connected with mesocoxa. The metathoracic venter is narrow, in front it takes the form of a thickened plate, surrounding metacoxae (cx3) (Figs 3A, B).

**Figure 4.**
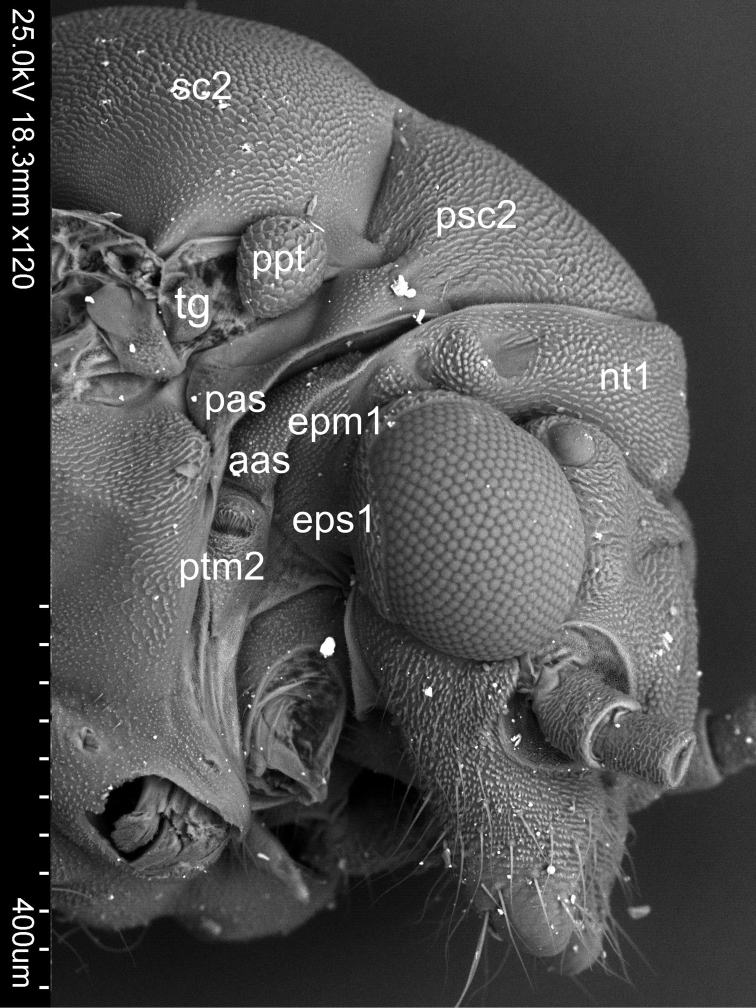
Sclerites between pleurites of pro- and mesothorax of *Cacopsylla crataegi*

## Discussion

The analysis of the thorax morphology of selected *Cacopsylla* species did not indicate any characters defining the subgenera described by Ossiannilsson (1970) and Loginova (1978). However, characters which are common for all *Cacopsylla* species are described. In the analysed species the pronotum is narrow, arcuate medially and well-defined laterally. The pronotum is narrower than the head including eyes, but wider than vertex; in all species it is laterally as long as it is along the midline. The pronotal shape is homogenous within this genus but differs from some other psyllid genera (Ouvrard et al. 2002, Drohojowska 2009a). Detailed studies on the thorax morphology were made by the senior author and the results will be published soon. Generally, a massive, wide and straight pronotum is observed in the Lividae family, narrower than the width of the head and slightly bended is observed in the Aphalaridae, Homotomidae and Phacopteronidae families. In the representatives of the Triozidae family one can observe a narrowing and bending of pronotum which modify it to become a thin, hardly visible batten. In the species of Carsidaridae the pronotum is wide, slightly bended, whereas in the case of Calyophyidae the pronotum is much bended and narrowing to a different extent in the middle part. In the species of the Psyllidae family the pronotum has a diversified shape (Drohojowska 2009a, Drohojowska unpublished data).

An additional feature common for all *Cacopsylla* species is the straight rather than undulating propleural sulcus stretching from the basal coxal appendix to hind corner of the pronotum, and not in the middle, like for example in *Craspedolepta* species or the fore corner of pronotum like in *Livia* species (Drohojowska 2009a, b). Three additional sclerites (aas, pas and peritrema) are well visible in all examined species. The shape of mesothoracic sclerites and sulcus is the same in all *Cacopsylla* species. Loginova (1978) suggested that the mesoscutum is distinctly longer than the mesopraescutum along the median longitudinal body axis and the mesopraescutum is about twice as long as the pronotum in *Thamnopsylla* species,whereas in the other subgenera the mesoscutum is as long as or slightly longer than the mesopraescutum and both are more than twice as long as the pronotum. We could not confirm this suggestion. An additional character common to all species is the shape of anapleural cleft. The position of the anapleural cleft (acl2) varies in psyllids ranging from diagonal to almost horizontal (Drohojowska 2009a) but in all *Cacopsylla* species it is straight and almost horizontal. The parapteron (ppt) and tegula (tg) have the same oval shape, the anterior pleural wing process (apwp) is thin and elastic, the metathorax stigma (stg3), trochantin on metathorax (trn3) and the external fossa of the trochantinal apodeme (ftna3) are well visible and the katepisternal complex (kes2) is rounded and striped in all *Cacopsylla* species. The connecting points of the mesopraescutum with the mesoscutum are symmetrical and onefolded for every species. Only in *Cacopsylla ambiqua* the points are double (Fig. 1B). It seems that it is a character specific to this species because this type of connection does not occur in other *Hepatopsylla* species and in other examined *Cacopsylla* species.

This and earlier studies (Journet and Vickery 1978, Drohojowska 2009b) suggest that the thorax morphology is relatively homogenous within a genus.

The study of thorax morphology of *Cacopsylla* species shows that the only difference between the male and the female is in the size of the latter. Shapes and proportions between particular elements of the thorax are the same in the representatives of both sexes. The results confirm those of previous studies (Drohojowska 2009b).

## References

[B1] Burckhardt D (2010) Identification key for the Central European *Cacopsylla* species. http://www.psyllidkey.eu

[B2] Burckhardt D Ouvrard D (2012) A revised classification of the jumping plant- lice (Hemiptera: Psylloidea). Zootaxa 3509: 1-34.

[B3] Crawford DL (1914) A monograph of the jumping plant- lice or Psyllidae of the new world. Smithsonian Institution United States National Museum Bulletin 85: 1-186. doi: 10.5479/si.03629236.85.1

[B4] Drohojowska J (2009a) General information on thorax morphology of selected species of psyllids (Hemiptera, Psylloidea). Monograph Aphids and Other Hemipterous Insects 15: 5-16.

[B5] Drohojowska J (2009b) Structure of head and thorax of *Livia juncorum* (Hemiptera, Psylloidea). Monograph Aphids and Other Hemipterous Insects 15: 17-30.

[B6] Heslop-Harrison G (1951) Subfamily separation in the Homopterous Psyllidae-II. Annals and Magazine of Naturel History 12 (4): 1-35.

[B7] Hodkinson ID White IM (1979) Homoptera Psylloidea. Handbooks for the Identification of British Insects 2: 1-98.

[B8] Hollis D (2004) Australian Psylloidea: Jumping Plantlice and Lerp Insects. Australian Biological Resources Study, Canberra, Australia, 216 pp.

[B9] Journet AR Vickery VR (1978) Studies on Nearctic Craspedolepta Enderlain, 1921 (Homoptera, Psylloidea): external morphology. Canadian Entomologist, 110: 13-36. doi: 10.4039/Ent11013-1

[B10] Klimaszewki SM (1975) Psyllodea- Koliszki (Insecta: Homoptera). Fauna Polski, Warszawa, 3: 1-294.

[B11] Li F (2011) Psyllidomorpha of China (Insecta: Hemiptera). Science Press, Beijing, China, 1976 pp.

[B12] Loginova MM (1978) Classification of the genus Psylla Geoffr. (Homoptera, Psyllidae). Entomologicheskoe Obozrenie 57: 808-824.

[B13] Ouvrard D Bourgoin T Campbell BC (2002) Comparative morphological assessment of the psyllid pleuron (Insecta, Hemiptera, Sternorrhyncha). Journal of morphology 252: 276-290. doi: 10.1002/jmor.1105 11948675

[B14] Ouvrard D Burckhardt D Soulier-Perkins A Bourgoin T (2008) Comparative morphological assessment and phylogenetic significance of the wing base articulation in Psylloidea (Insecta, Hemiptera, Sternorrhyncha). Zoomorphology 127: 37-47. doi: 10.1007/s00435-007-0049-x

[B15] Ossiannilsson F (1970) Contributions to the Knowledge of Swedish Psyllids (Hem. Psylloidea) 1-4. Entomologica scandinavica, 1: 135-144.

[B16] Ossiannilsson F (1992) The Psylloidea (Homoptera) of Fennoscandia and Danmark. Fauna entomologica Scandinavica 26. E. J. Brill, Leiden- New York- Köln, 346 pp.

[B17] Stough HB (1910) The hackberry Psylla, *Pachypsylla celtidismammae* Riley. A study in comparative morphology. Kansas University Science Bulletin 5: 121-165.

[B18] Taylor LH (1918) The thoracic sclerites of Hemiptera and Heteroptera. Annals of the Entomological Society of America 11: 225-254.

[B19] Tremblay E (1965) Studio morfo- biologico sulla *Trioza tremblayi* Wagner (Hemiptera- Homoptera, Psyllidae). Bolletino del Laboratorio di Entomological Agraria Filippo Silvestri 23: 37–138.

[B20] Weber H (1929) Kopf und Thorax von Psylla mali Schmidberger (Hemiptera- Homoptera). Eine morphogenetische Studie. Zeitschrift für Morphologie und Ökologie der Tiere 14: 59–165. doi: 10.1007/BF00419345

